# Relationship between health-related quality of life, perceived family support and unmet health needs in adult patients with multimorbidity attending primary care in Portugal: a multicentre cross-sectional study

**DOI:** 10.1186/s12955-016-0559-7

**Published:** 2016-11-11

**Authors:** Filipe Prazeres, Luiz Santiago

**Affiliations:** 1 Faculdade de Ciências da Saúde, Universidade da Beira Interior, Covilhã, 6200-506 Portugal; 2 Centro de Saúde de Aveiro, Aveiro, 3810-000 Portugal; 3 USF Topázio, Coimbra, 3020-171 Portugal

**Keywords:** Multimorbidity, Health-related quality of life, Family support, Unmet health needs, Primary health care, Portugal

## Abstract

**Background:**

Multimorbidity has a high prevalence in the primary care context and it is frequently associated with worse health-related quality of life (HRQoL). Few studies evaluated the variables that could have a potential effect on HRQoL of primary care patients with multimorbidity. The purpose of this study, the first of its kind ever undertaken in Portugal, is to analyse the relationship between multimorbidity, health-related quality of life, perceived family support and unmet health needs in adult patients attending primary care.

**Methods:**

Multicentre, cross-sectional survey conducted among primary care patients with multimorbidity. It included 521 participants (64.1 % females) who met the inclusion criteria. HRQoL was evaluated using the Portuguese Short Form-12 Health Status Questionnaire. The Portuguese Family APGAR was used to measure the perceived family support. A patients’ unmet health needs questionnaire was used. The unmet needs for medical, surgical and dental care; prescription medications; mental healthcare or counselling; and eyeglasses or other technical aid was assessed. Descriptive and multivariate analyses were performed.

**Results:**

The sample had an overall average of 4.5 chronic health problems. Increased multimorbidity levels were linked to worse health-related quality of life, particularly the physical health. Some variables were confirmed as playing a role on health-related quality of life. Male patients with high monthly incomes and highly functional families had better physical and mental health. High levels of education and the presence of asthma were also associated with better physical health. Contrariwise, elderly patients with high levels of multimorbidity and with osteoarthritis had lower physical health. The majority of the patients did not have unmet health needs. When health needs were stated they were mostly for generalist medical care, dental care, and eyeglasses/other technical aid. Financial insufficiency was the primary reason for not fulfilling their health needs.

**Conclusion:**

To improve the quality of life of multimorbid patients, within primary care practices and health delivery systems, one should take into special account the sex of the patient, the perceived family support and the self-perceived economic status because of their relationship with both physical and mental health. Limitations and recommendations are discussed.

## Background

The prevalence of multimorbidity, defined as the co-occurrence of 2 or more chronic health problems within one person [1], is increasing worldwide due to the effects of improved living conditions, better medical care and an aging population [2, 3]. Portuguese epidemiologic data follows the same tendency, with a high prevalence of multimorbidity (72.7 %) amongst adult patients attending primary care [4]. Factors such as social deprivation [5], marginalisation [6], mental health disorders [5], and poor housing conditions [7] are associated with an increased prevalence of multimorbidity.

Living with multimorbidity can be a difficult task for the patients as well as for their healthcare providers. Multimorbid patients are more likely to die early [8], experience poor clinical outcomes [9] and a decline of physical functioning [10]. When describing the health burden of chronic diseases, healthcare providers should include measurements of health-related quality of life (HRQoL) [11].

HRQoL is a multidimensional concept that includes domains related to physical, mental, emotional and social functioning associated with an illness or treatment [12]. The Centers for Disease Control and Prevention (CDC) defined HRQoL as an individual’s or group’s perceived physical and mental health over time [13]. Self-rated health status is also a predictor of mortality [14].

Since multimorbidity has a significant negative impact on HRQoL [15–19], it would be expected that this relationship would be commonly researched, particularly in the primary care context where the majority of treated patients are multimorbid [20], but this is not the case [16]. Limited information exists about the influence of sociodemographic factors (e.g. social support, educational background, economic status) on HRQoL of primary care patients with multimorbidity [2]. There is some evidence to suggest that strong social support from family members can protect against illness or disability [21] and improve chronic illness outcomes [22].

Therefore, a comprehensive approach to the multimorbid patient should take into consideration not only the measurement of HRQoL, but also the impact of the different sociodemographic factors on HRQoL [2, 16], including family support, and the health needs of this group of patients, for GPs to improve care to multimorbid patients and ultimately improve the efficacy of healthcare planning and deal with the inherent social costs, particularly in contexts with limited resources [23].

The purpose of the present study, the first of its kind ever undertaken in Portugal, is to determine the impact of multimorbidity on HRQoL in patients aged 18 years and above attending primary care. Specifically, this study aims to i) characterise the unmet health needs of adult patients with multimorbidity, ii) assess family support to adult patients with multimorbidity; iii) analyse the relationship between multimorbidity, patients’ health-related quality of life, perceived family support and unmet health needs.

## Methods

### Study design

A multicentre, cross-sectional survey was conducted among primary care patients with multimorbidity in thirteen Primary Care Centres in the Centre region of Portugal, between January 2014 and January 2015. This study reports on Phase II of a Three-Phase project (MM-PT: Multimorbidity in primary care in PorTugal) aimed at raising awareness on the relevance to deal with multimorbidity in Portugal. Details regarding the full project’s protocol were previously published elsewhere [24].

The study was approved by local research ethics committees (Faculty of Health Sciences – University of Beira Interior – and the Central Health Region of Portugal) and was conducted in accordance with the principles of the Declaration of Helsinki [25]. Informed consent was obtained from all participants and confidentiality was maintained.

The reporting of this study conforms to the Strengthening the Reporting of Observational Studies in Epidemiology (STROBE) statement [26].

### Sample

Study size and sampling of the Primary Care Centres have been described elsewhere [24]. Enrolled GPs recruited patients presenting for a primary care appointment at each of the institutions during the period of the study. They ensured that each individual patient met the criteria for eligibility. Participation inclusion criteria included being a willing volunteer to participate; being 18 or more years of age; and having a recorded diagnosis of at least two chronic health problems, of which at least one was required to be hypertension, diabetes, asthma or osteoarthritis. These 4 diseases were selected because, on the one hand, they have high prevalence and are frequently associated with low quality of life, and on the other hand, there are national efforts to implement specific recommendations for the diagnosis, treatment and control of these diseases [24]. Exclusion criteria included being acutely unwell or presenting inability to provide independent informed consent. A total of 548 patients were approached (including approximately 10 % above estimated sample size to account for expected missing data). Twenty-seven individuals refused to participate without any stated reason. Therefore, 521 interviews were performed; all interviews were fully completed and so no missing data was encountered.

### Data collection procedures

Data collection was performed by protocol [24]. It was carried out through a structured face-to-face interview delivered by the investigator or a GP/GP trainee. In order to minimize interview bias, all interviewers were very experienced in conducting face-to-face interviews and, if needed, received additional training from members of the research team. Interviews were performed after the patient’s clinical visit or while waiting for their appointment. Consenting patients were evaluated at a single-time point and the responses were recorded on paper. The average time of the interview was 15 min.

### Measures

#### Sociodemographic characteristics

Using the personal information section of the Portuguese version of the EASY-Care questionnaire [27], self-reported data were obtained for sex (male/female), age group (18-34/35-49/50-64/≥65 years), residence area (urban/rural), current marital status (married-cohabiting/single/widowed/separated-divorced), number of years of formal education (less than 6 years/at least 6 but not more than 9 years/more than 9 years), living arrangements (couple/extended family/alone/other), professional status (pensioner-retired/employed/unemployed/housewife/student) and self-perceived economic status (“Just enough to make ends meet”/“Not enough to make ends meet”/“Some money left over”).

#### Medical history and measures of multimorbidity

Patients’ chronic health problems were collected by the investigator or a GP/GP trainee using 3 data sources for each patient: GPs knowledge of patient’s history, patient’s self-report and medical records.

The current study considered the 147 International Classification of Primary Care (ICPC-2) diagnoses gathered by O’Halloran et al. [28] (Family Medicine Research Centre, University of Sydney). These diagnoses were defined as chronic by the O’Halloran criteria: a) have a duration that has lasted, or is expected to last, at least 6 months; b) have a pattern of recurrence, or deterioration; c) have a poor prognosis and d) produce consequences, or sequelae that impact on the individual’s quality of life [28].

Multimorbidity was measured based on simple counts of chronic health problems coexisting within one person. Drawing on the categorization of Kadam et al. [29], multimorbidity was classified into low morbidity count (2 or 3 chronic health problems), medium (4 or 5 chronic health problems) and high (6 or more chronic health problems). No assessment of disease severity in the multimorbid conditions found was undertaken.

#### Health-related quality of life

The Portuguese Short Form-12 Health Status Questionnaire (SF-12) [30], was used to assess health-related quality of life from the patient’s perspective.

The SF-12 [31] is a short form survey with 12 questions. In studies with large samples (*n =* 500) it is a valid alternative to the 36-item Short Form (SF-36) [31] since it takes an average of 2 min to administer and has a reduced respondent and administrative burdens [31–33]. The SF-12 addresses the same 8 domains as identified in the SF-36: physical functioning (PF, 2 items); role limitations due to physical health problems (RP, 2 items); bodily pain (BP, 1 item); general health perceptions (GH, 1 item); vitality (VT, 1 item); social functioning (SF, 1 item); role limitations due to emotional problems (RE, 2 items) and mental health (MH, 2 items). The SF-12 also assesses 2 health status composite scores: physical health (Physical Component Summary, PCS) and mental health (Mental Component Summary, MCS). These composite scores are generated using an algorithm for comparison to normative data—general United States (US) population—with a mean score of 50 and a standard deviation of 10; scores above 50 indicate better physical or mental health and scores bellow 50 indicate worse health [31]. Since there is little difference between standard scoring algorithms (US-derived) and country-specific algorithms, the use of the standard scoring algorithms is recommended to allow data comparisons across countries [34].

This brief tool (SF-12) has been used extensively in clinical and population-based studies [32], including those with chronic health conditions. The Portuguese version has shown satisfactory reliability and validity [30]. In the present study, both summary measures exceeded the 0.70 level for Cronbach’s Alpha (internal consistency) indicating satisfactory results (α for the PCS and the MCS was 0.84 and 0.82, respectively).

#### Family support

The Portuguese Family APGAR Questionnaire [35, 36] was used to measure the perceived family support of patients with multimorbidity. This questionnaire is commonly used in the Portuguese primary care setting, since it is widely available to GPs as an integral part of the electronic health records software*.*

Family APGAR Questionnaire features five closed-ended questions measuring family member’s satisfaction with each of the five basic components of family function (Adaptation, Partnership, Growth, Affection and Resolve) [37]. The response format is a three-point scale (“almost always”—two points; “some of the time”—one point; or “hardly ever”—zero points). The scores for each of the five questions after totaled originate the following categories: a) severely dysfunctional families (0 to 3 points); b) moderately dysfunctional families (4 to 7 points); or c) highly functional families (8 to 10 points) [37, 38]. In the study, Cronbach’s Alpha (internal consistency) for the total scale was 0.86.

#### Patients’ unmet health needs

The unmet needs for medical, surgical and dental care; prescription medications; mental healthcare or counselling; and eyeglasses or other technical aid were evaluated. The detailed set of questions used in this study are provided in the previously published protocol [24]. These questions were pilot tested for comprehensibility in 50 adult general practice patients, no changes were necessary.

### Statistical analyses

Variables were summarized using descriptive statistics namely absolute (n) and relative (%) frequencies for categorical variables and mean and standard deviation (SD) for numerical variables.

Univariate analyses were performed to study the association between presence of unmet health needs, presence of moderate/severe dysfunctional family and health related quality of life with patients’ characteristics using Chi-square test (categorical variables) or Kruskal-Wallis test (numerical variables which did not follow normal distribution).

Multiple binary logistic regression for presence of unmet health needs and perceived moderately/severely dysfunctional family was performed using variables found to be statistically significant in the univariate analysis and a stepwise selection method (variables were entered considering a stepwise probability of 0.05). Pairwise comparisons within comorbidity groups were performed using Dunn’s [39] procedure with a Bonferroni correction for multiple comparisons.

Multiple linear regression was performed for PCS and MCS scores using variables significant in the univariate analysis and a stepwise selection method (variables were entered considering a stepwise probability of 0.05).

All tests were two-sided considering a significance level of 0.05. Statistical analysis was performed using IBM SPSS Statistics for Windows, Version 21.0 (IBM Corporation, Armonk, NY, USA).

## Results

### Characteristics of participants

Demographic and medical characteristics of the 521 study participants are shown in Table 1. Mean age was 58.2 years (61.2 years for men and 56.6 years for women). The majority of participants were female (64.1 %) and 57.2 % had a low level of education. Approximately half of those surveyed (46.3 %) reported a sufficient monthly income. Seventy per cent were married or cohabiting, and 54.3 % lived as a couple.

**Table 1:** Demographic and medical characteristics of participants (*n =* 521)

Sex, % (*n*)	
Women	64.1 (334)
Men	35.9 (187)
Age group, % (*n*)	
18–34 years	9.0 (47)
35–49 years	15.7 (82)
50–64 years	39.5 (206)
≥ 65 years	35.7 (186)
Residence area, % (*n*)	
Urban	49.1 (256)
Rural	50.9 (265)
Marital status, % (*n*)	
Married/cohabiting	70.2 (366)
Single	11.5 (60)
Widowed	8.6 (45)
Separated/divorced	9.6 (50)
Living arrangements, % (*n*)	
Couple	54.3 (283)
Extended Family	31.3 (163)
Alone	11.9 (62)
Other (including care home)	2.5 (13)
Education, % (*n*)	
Low level (less than 6 years)	57.2 (298)
Medium level (at least 6 but not more than 9 years)	19.4 (101)
High level (more than 9 years)	23.4 (122)
Professional status, % (*n*)	
Pensioner/retired	43.0 (224)
Employed (full-time/part time)	34.2 (178)
Unemployed	11.3 (59)
Housewife	10.4 (54)
Student	1.2 (6)
Monthly income, % (*n*)	
“Not enough to make ends meet”	38.2 (199)
“Just enough to make ends meet”	46.3 (241)
“Some money left over”	15.5 (81)
Multimorbidity group, % (*n*)	
Low (2–3 chronic problems)	42.2 (220)
Medium (4–5 chronic problems)	27.6 (144)
High (≥6 chronic problems)	30.1 (157)
Chronic health problems^a^, % (*n*)	
Hypertension	61.8 (322)
Diabetes mellitus	29.0 (151)
Asthma	17.3 (90)
Osteoarthritis	57.6 (300)
Other prevalent chronic health problems^a^, % (*n*)	
Lipid disorder	63.1 (329)
Depressive disorder	19.6 (102)
Obesity	14.2 (74)
Overweight	10.9 (57)
Varicose veins of leg	9.8 (51)
Benign prostatic hypertrophy	8.1 (42)
Osteoporosis	7.9 (41)
Goitre	7.7 (40)
Liver disease	7.1 (37)
Anxiety disorder/anxiety state	5.4 (28)

^a^The same participant may have more than one condition

Low morbidity count was present in 42.2 % of the sample, 27.6 % had a medium morbidity count and a high morbidity count was encountered in 30.1 %, with an overall average of 4.5 chronic health problems per participant (4.6 in men and 4.5 in women).

### Unmet health needs and multimorbidity

Unmet health needs are described in Table 2. At least one unmet health need in the preceding 12 months was reported by about one third of the patients and 7.3 % reported two or more unmet health needs.

**Table 2:** Unmet health needs, perceived family support and health related quality of life

	Overall [*n =* 521]	Multimorbidity Group			
		Low [*n =* 220]	Medium [*n =* 144]	High [*n =* 157]	*P-*value
No. of unmet needs by participant, % (*n*)					
0	69.1 (360)	69.1 (152)	66.7 (96)	71.3 (112)	0.676
1	23.6 (123)	23.2 (51)	25.7 (37)	22.3 (35)	
2	6.0 (31)	6.8 (15)	5.6 (8)	5.1 (8)	
3	0.8 (4)	0.5 (1)	0.7 (1)	1.3 (2)	
4	0.6 (3)	0.5 (1)	1.4 (2)	0.0 (0)	
Type of unmet needs, % (*n*)^a^					
Prescription medications	1.2 (6)	0.9 (2)	2.8 (4)	0.0 (0)	n.a.
General medical care	13.1 (68)	13.2 (29)	16.0 (23)	10.2 (16)	0.330
Surgical care	1.0 (5)	0.5 (1)	1.4 (2)	1.3 (2)	n.a.
Mental healthcare/counselling	1.0 (5)	0.0 (0)	2.8 (4)	0.6 (1)	n.a.
Dental care	12.7 (66)	13.2 (29)	11.1 (16)	13.4 (21)	0.803
Eyeglasses/technical aid	11.3 (59)	12.3 (27)	10.4 (15)	10.8 (17)	0.838
Reasons, % (*n*)^a^					
Professional	3.1 (16)	5.5 (12)	2.8 (4)	0.0 (0)	n.a.
Too sick	1.2 (6)	1.4 (3)	1.4 (2)	0.6 (1)	n.a.
Mobility	1.7 (9)	0.5 (1)	2.8 (4)	2.5 (4)	n.a.
Care provider of a dependent	1.2 (6)	1.4 (3)	0.7 (1)	1.3 (2)	n.a.
Afraid to leave home	1.0 (5)	0.5 (1)	2.8 (4)	0.0 (0)	n.a.
Other concerns	1.0 (5)	1.8 (4)	0.7 (1)	0.0 (0)	n.a.
Financial	18.0 (94)	18.2 (40)	16.7 (24)	19.1 (30)	0.857
Access to GP consultations	8.6 (45)	6.4 (14)	12.5 (18)	8.3 (13)	0.123
Family APGAR categories, % (*n*)					
Severely Dysfunctional	9.2 (48)	7.3 (16)	8.3 (12)	12.7 (20)	0.363
Moderately Dysfunctional	20.3 (106)	22.3 (49)	18.1 (26)	19.7 (31)	
Highly Functional	70.4 (367)	70.5 (155)	73.6 (106)	67.5 (106)	
SF-12 scores, mean (SD)					
PF	44.2 (12.4)	48.5 (10.8)	42.4 (12.4)	39.7 (12.6)	<0.001
RP	43.1 (12.9)	47.2 (11.1)	42.1 (13.0)	38.1 (13.2)	<0.001
BP	40.0 (12.4)	43.6 (12.0)	39.1 (12.6)	36.0 (11.5)	<0.001
GH	34.6 (11.2)	39.0 (11.5)	32.5 (9.9)	30.4 (9.6)	<0.001
VT	48.0 (11.4)	50.3 (10.8)	47.1 (11.3)	45.7 (11.6)	<0.001
SF	44.8 (13.5)	47.5 (12.7)	43.8 (13.5)	41.8 (14.1)	<0.001
RE	44.1 (12.6)	46.3 (11.3)	44.2 (12.8)	40.8 (13.5)	<0.001
MH	45.1 (12.9)	46.9 (12.5)	45.5 (12.7)	42.3 (13.1)	0.002
PCS	40.3 (11.9)	45.0 (11.0)	38.3 (11.3)	35.5 (11.1)	<0.001
MCS	46.6 (12.2)	47.8 (12.0)	47.0 (12.3)	44.6 (12.4)	0.033

n.a. – Chi-square test not applicable due to low frequencies

*PF* physical functioning, *RP* role physical, *BP* bodily pain, *GH* general health, *VT* vitality, *SF* social functioning, *RE* role emotional, *MH* mental health, *PCS* physical component summary, *MCS* mental component summary

^a^The same participant may have reported more than one option

The most common unmet health needs were related to generalist medical care, dental care, and eyeglasses or other technical aid. The most frequently cited reason for explaining the presence of unmet health needs was financial (18 % of the respondents had to spend their money for food, clothing, housing, etc.).

Presence of unmet health needs was statistically similar across the three multimorbidity groups (*p =* 0.676) (Table 2).

### Unmet health needs and other characteristics

From univariate analysis, presence of unmet needs was more frequently reported by women than men (37.7 % vs. 18.7 %; *p <* 0.001), by patients with lower/medium education levels than higher level (33.3 % vs. 23.0 %; *p =* 0.030), by patients with insufficient monthly income than by sufficient/higher monthly incomes (48.2 % vs. 22.4 %/13.6 %; *p <* 0.001), by non-diabetics than diabetic patients (33.8 % vs. 23.8 %; *p =* 0.026), and by patients with osteoarthritis than without it (35.7 % vs. 24.4 %; *p =* 0.006). Moreover, patients reporting unmet health needs were 5 years younger than patients without unmet needs (average/range: 55 years/20–92 years vs. 60 years/18–93 years; *p =* 0.003).

Multivariate analysis (Table 3) shows that variables remaining important in explaining the presence of unmet health needs were sex, age, monthly income and education level. Women were 2.3 times more likely to report unmet health needs than men. Patients aged 18–34 years were 2.5 times more likely to report unmet health needs than older patients. Patients with insufficient monthly income were nearly 3.3 times more likely to report unmet health needs. Patients with low/medium level of education were 2 times more likely to report unmet health needs. The presence of diabetes or osteoarthritis was not statistically significant to the model.

**Table 3:** Multiple logistic regression for presence of unmet health needs

Factors	OR (95 % CI)	*P-*Value
Sex		
Women	2.33 (1.48–3.66)	<0.001
Age group		
18–34 years	2.47 (1.21–5.05)	0.013
Monthly income		
“Not enough to make ends meet”	3.29 (2.17–4.99)	<0.001
Education		
Low/medium level	2.03 (1.15–3.58)	0.015

Reference category: sex = male; monthly income = “Just enough to make ends meet” aggregated with “Some money left over”; age grou*p =* higher than 34 years; education = high level

### Perceived family support and multimorbidity

Regarding the family support as reported by the sample, the majority (70.4 %) perceived their families to be highly functional, 20.3 % reported as being moderately dysfunctional and 9.2 % severely dysfunctional (Table 2). On a scale of 0 to 10 (where 0 corresponds to the lowest and 10 to the highest family support) this represents a mean (SD) of 7.9 (2.7) for the overall sample.

According to the multimorbidity range, the group of patients with a high morbidity count (6 or more chronic health problems) had slightly higher perception of having a dysfunctional family than the low and medium multimorbidity groups; although this difference was not statistically significant (*p =* 0.363) (Table 2).

### Perceived family support and other characteristics

From univariate analysis, perception of family dysfunction (moderate/severe) was more frequently reported by women than men (35.6 % vs. 18.7 %; *p <* 0.001), by patients living in urban that rural areas (33.6 % vs. 25.7 %; *p =* 0.047), by not married than married patients (45.2 % vs. 23.0 %; *p <* 0.001), by patients with insufficient monthly income than with sufficient/higher monthly incomes (40.7 % vs. 25.3 %/14.8 %; *p <* 0.001), by patients living alone than with an extended family (54.8 % vs. 22.7 %, *p <* 0.001) and by patients with unmet health needs than without them (44.1 % vs. 23.1 %; *p <* 0.001).

Table 4 shows that variables remaining associated with perception of family dysfunction in multivariate analysis were sex, marital status, monthly income, living arrangements and presence of unmet needs. Women were 2 times more likely to perceive a dysfunctional family than men. Single/divorced/widow patients were 2.8 times more likely to perceive a dysfunctional family than married patients. Patients with insufficient monthly income were 1.8 times more likely to perceive a dysfunctional family. Patients with unmet needs were 1.9 times more likely to report a dysfunctional family. Patients living alone are more likely to perceive a dysfunctional family than patients living in an extended family.

**Table 4:** Multiple logistic regression for perceived moderately/severely dysfunctional family

Factors	OR (95 % CI)	*P-*Value
Sex		
Women	2.01 (1.26–3.20)	0.003
Marital status		
Single/divorced/widow	2.77 (1.48–5.17)	0.001
Monthly income		
“Not enough to make ends meet”	1.81 (1.18–2.78)	0.007
Living arrangements		
Couple	0.905 (0.39–2.09)	0.815
Extended family	0.389 (0.19–0.79)	0.009
Other	0.910 (0.26–3.22)	0.884
Unmet needs		
Presence	1.94 (1.24–3.0)	0.003

Reference category: sex = male; marital status = married; living arrangements = alone; monthly income = “Just enough to make ends meet” aggregated with “Some money left over”; unmet needs = absent

### Health-related quality of life and multimorbidity

**Fig. 1:**
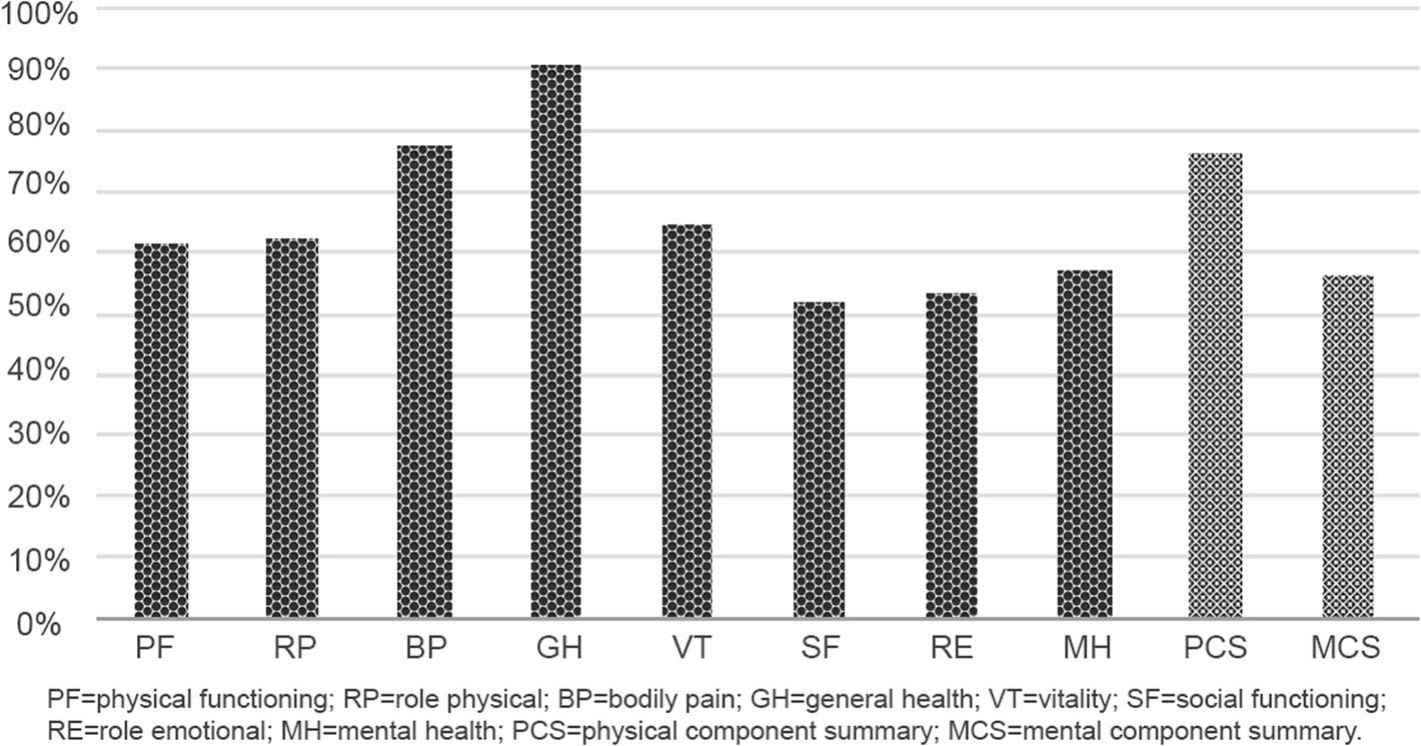
Percentage of patients with SF-12 scores less than 50

The majority of the sample had a score bellow 50 (mean of the reference population) in all eight domains of the SF-12, particularly in general health (91.0 %) and bodily pain (77.9 %) (Fig. 1). Regarding the two health status composite scores, physical health (PCS) was worse than the mental health (MCS) (Table 2).

All SF-12 scores were statistically related with the multimorbidity groups, showing that health related quality of life decreases when levels of multimorbidity rise (Table 2). Pairwise comparisons revealed no statistically differences between (a) medium versus high morbidity groups in PF (*p =* 0.196), BP (*p =* 0.063), GH (*p =* 0.226), VT (*p =* 0.928), SF (*p =* 0.799) and PCS scores (*p =* 0.139); (b) medium versus low/high in RE, MH and MCS scores.

### Health-related quality of life and other characteristics

Univariate analysis shows that the SF-12 eight domains and the two health status composite scores were related to at least one participant characteristic besides the multimorbidity level (Table 5). Indeed, all SF-12 scores were statistically related with marital status, monthly income, perceived family support (family APGAR) and the presence of unmet needs (Table 5).

**Table 5:** Association between SF-12 scores and participants’ characteristics

Characteristic	SF-12 scores (*P-*values*)									
	PF	RP	BP	GH	VT	SF	RE	MH	PCS	MCS
Sex	<0.001	0.023	<0.001	0.016	0.004	<0.001	n.s	<0.001	0.001	<0.001
Age group	<0.001	<0.001	0.001	<0.001	n.s	n.s	n.s	n.s	<0.001	n.s.
Living arrangements	0.010	0.009	n.s	n.s	n.s	0.025	n.s	0.012	0.007	0.043
Education	<0.001	<0.001	<0.001	<0.001	<0.001	0.019	n.s	0.017	<0.001	n.s.
Residence area	n.s.	n.s	n.s	0.027	n.s	0.045	n.s	n.s	n.s.	n.s.
Marital status	<0.001	0.001	0.001	<0.001	0.017	<0.001	<0.001	0.003	<0.001	0.001
Monthly income	<0.001	<0.001	<0.001	<0.001	<0.001	<0.001	<0.001	<0.001	<0.001	<0.001
Professional status	<0.001	<0.001	<0.001	<0.001	0.021	n.s	0.012	0.011	<0.001	n.s.
Family APGAR	<0.001	<0.001	<0.001	<0.001	<0.001	<0.001	<0.001	<0.001	<0.001	<0.001
Unmet health needs	<0.001	0.003	<0.001	<0.001	0.002	<0.001	<0.001	<0.001	0.001	<0.001
Asthma	<0.001	<0.001	<0.001	<0.001	n.s	n.s	n.s	n.s	<0.001	n.s.
Osteoarthritis	<0.001	<0.001	<0.001	<0.001	0.002	n.s	n.s	0.023	<0.001	n.s.
Diabetes	0.002	0.047	n.s	<0.001	n.s	n.s	n.s	n.s	0.001	n.s.
Hypertension	<0.001	0.001	0.029	<0.001	n.s	n.s	n.s	n.s	<0.001	n.s.

*PF* physical functioning, *RP* role physical, *BP* bodily pain, *GH* general health, *VT* vitality, *SF* social functioning, *RE* role emotional, *MH* mental health, *PCS* physical component summary, *MCS* mental component summary

*P-values are for score comparison between categories of each characteristic (bivariate analysis)

Multivariate analysis for PCS scores (Table 6) shows that sex, age, monthly income, education, multimorbidity groups, family APGAR, osteoarthritis and asthma were statistically significant predictors for PCS score. Male, higher monthly income, higher level of family functionality, higher level of education, younger age, lower levels of multimorbidity, absence of osteoarthritis, and presence of asthma are related with a predicted higher score at PCS. Regarding MCS scores, sex, monthly income, and family APGAR are statistically significant predictors. Being male, having a higher monthly income and a higher level of family functionality are MCS protective factors.

**Table 6:** Multiple linear regression for PCS and MCS

	PCS			MCS		
Variable	*B*	*SE*_*B*_	β	*B*	*SE*_*B*_	β
Intercept	34.82	3.49	-	22.02	2.41	-
Sex	3.23	0.97	0.13*	2.44	1.03	0.10*
Age group	−1.73	0.65	−0.14*	-	-	-
Monthly Income	1.71	0.67	0.10*	2.92	0.71	0.17*
Education	2.28	0.68	0.16*	-	-	-
Multimorbidity group	−2.41	0.60	−0.17*	-	-	-
Family APGAR	1.93	0.71	0.11*	6.16	0.78	0.33*
Osteoarthritis	−2.76	0.98	−0.12*	-	-	-
Asthma	3.23	1.34	0.10*	-	-	-

*SE*_*B*_ standard error of the coefficient*, β* = standardized coefficient

*p < 0.05; B = unstandardized regression coefficient

## Discussion

The current study represents the first analysis on health-related quality of life among adult patients with multimorbidity in a primary-care context in Portugal.

Globally, the multimorbid sample in this study reported poorer health-related quality of life than the reference population (recommended for international comparisons) [34], which demonstrates the adverse effect of multimorbidity on health-related quality of life. This overall finding is in line with the available literature [15–19, 40]. However, existing studies lack comparable samples and methodologies and no direct comparisons can be made [2].

Health-related quality of life decreased inversely with the number of concurrent chronic health problems, which reflects previous studies [16, 18, 41, 42]. This occurrence was particularly evident when comparing the low (2 or 3 chronic health problems) and high (6 or more chronic health problems) morbidity count groups for all SF-12 dimensions (the eight domains and the two health status composite scores). Nonetheless, there was only a moderate evidence of the effect of multimorbidity on mental health compared to its effect on physical health, which also seems to be consistent with previous research [16, 17].

As could be expected from previous studies [43–45], increasing age was associated with poorer physical health. However, no effect of aging was observed on mental health. This discrepancy can be attributed to the psychological adaptation to illness, over time [46].

Female sex [2, 42, 43, 47, 48], low level of education [2, 43, 49], and a low income [15, 49, 50] are commonly associated with impaired health-related quality of life, and the current study’s findings corroborate this. An implication of this is the possibility that multimorbid patients may benefit from financial aid through social policy programs.

This study also considered other variables that were earlier pointed out as having a possible impact on health-related quality of life [16] such as marital status, living arrangements and professional status. The current results do not show a clear relationship between these variables and health-related quality of life. Therefore, further work is still required to clarify the full impact of sociodemographic data on health-related quality of life in patients with multimorbidity [16].

Prior studies have noted the relationship between family APGAR scores and the presence of chronic illness [51, 52]. Despite its multiple chronic health problems, the study’s sample reported high family support. Family dysfunction was present at a quite lower proportion than in previous reports [52–54]. This inconsistency may be due to the fact that in previous studies the age of the sample was limited to the geriatric population whereas in this study the age group was 18+ years old. The established distribution of family support was the same between age groups (*p =* 0.182).

In this study, as expected by previous findings [55, 56], perceived family support had an impact on health-related quality of life. Multimorbid patients from dysfunctional families reported worse physical and mental health. From these results, it is possible to infer that adult patients with multimorbidity in a primary-care context may have a potential gain in health-related quality of life if family members provide support for their care. In Portugal, most of the support comes from families, more than three quarters of informal caregivers provide daily care [57]. Increased social support from family members improves chronic illness outcomes [22] (e.g. better glycaemic control for diabetic patients [58], better blood pressure control for hypertensive patients [59], and lower disease activity for patients with arthritis [60]). As such, GPs should devise efforts to inform and engage patients’ families as partners in the care of the multimorbid patient, notably the women living alone and with an insufficient monthly income.

In the present study, patients with unmet health needs had a statistically significant higher perception of having a dysfunctional family than those without unmet needs. The presence of unmet needs was also associated with lower health-related quality of life. Hence, family intervention programs for multimorbid patients (especially young women with an insufficient monthly income, living alone, and with low/medium level of education) will have to address their needs as to have a significant impact on quality of life and health outcomes [61].

Contrary to expectations, by taking into consideration the sample’s morbidity levels, the majority of the patients did not have unmet health needs. But when health needs were stated they were mostly for generalist medical care, dental care, and eyeglasses or other technical aid. Financial insufficiency was the primary reason for not fulfilling their health needs. These findings not only reinforce the previously stated necessity of financial support to multimorbid patients (in particular women), but also that primary care teams should organize resources and schedules to meet the medical care needs of multimorbid patients. Interestingly, younger patients reported greater unmet health needs than older patients. A possible explanation for this finding is the relationship of multimorbidity with higher out-of-pocket spending [62]. Portugal is among the four Organization for Economic Co-operation and Development (OECD) countries with the highest out-of-pocket spending, mostly due to the recently imposed restrictions on tax-deductible expenses [63]. This increase in expenditures affects younger taxpayers and leaves out the older poor patients with tax exemptions. A note of caution is due here, since patients’ needs may change as a result of the phase of illness, during major events, periods of disease exacerbation and patient’s socioeconomic status. Future studies with a longitudinal approach are therefore recommended.

Several limitations need to be acknowledged. Four chronic health problems have been selected based on their importance and although this excluded patients with multimorbidity without at least one of the selected conditions, the study’s sample captured 109 out of a total of 147 possible chronic health problems, a much higher number than the majority of the previously published health-related quality of life studies [16]. The current study did not take into account the severity of each chronic health problem and it had a cross-sectional design, so it was not possible to establish causal relationships. A sample selection bias due to the possibility of non-consecutive recruitment of patients by the GPs, should also be considered.

## Conclusion

The findings of this study link the increased multimorbidity levels to worse health-related quality of life, particularly the physical health, in multimorbid patients aged 18 and older attending primary care consultations. Some variables were confirmed as playing a role on health-related quality of life. As a result, to improve the quality of life of multimorbid patients, within primary care practices and health delivery systems, one should take into special account the sex of the patient, the perceived family support and the self-perceived economic status because of their relationship with both physical and mental health. This will also be of relevance when planning longitudinal and interventional studies regarding health-related quality of life.

Further research is suggested on larger nationwide samples to corroborate the results of the current study. It is also recommended to include the quality of household and living conditions in future health-related quality of life studies in the area.
